# Scrolling into hope and anxiety: a qualitative study of how short-video platforms shape psychological adjustment among Chinese parents of children with autism

**DOI:** 10.3389/fpsyg.2026.1952061

**Published:** 2026-09-09

**Authors:** Zijing Cun, Yutong Ye

**Affiliations:** 1 Yunnan University of Finance and Economics, Kunming, China; 2 Assumption University, Bangkok, Thailand

**Keywords:** autism spectrum disorder, parents, psychological adjustment, qualitative research, short-video platforms

## Abstract

**Background:**

Short-video platforms have become important sources of autism-related information, intervention guidance, and emotional support for parents of children with autism. However, algorithmic recommendations, selective presentation, and heterogeneous content may also heighten anxiety and complicate caregiving decisions. This study explored how short-video platforms shape the psychological adjustment of Chinese parents of children with autism.

**Methods:**

This study employed an exploratory qualitative design. Twenty-four parents (13 mothers and 11 fathers) were recruited using purposive and snowball sampling. Semi-structured, in-depth interviews lasting 45–75 min were conducted. Data were analyzed using thematic analysis, with social support theory and social comparison theory used as complementary interpretive frameworks.

**Results:**

Three themes and 12 subthemes were identified. Through audiovisual demonstrations, algorithmic recommendations, parent narratives, and longitudinal records of children’s development, short videos provided informational support, emotional resonance, and hope. Conversely, decontextualized intervention advice, repeated recommendations, selective success narratives, urgency-based messaging, and commercial promotion contributed to informational confusion, comparisons of children’s development and parental effort, self-doubt, and intervention-related anxiety. With increasing platform experience, parents recalibrated credibility judgments, comparison standards, emotional boundaries, and caregiving decisions by integrating online information with professional advice and their children’s individual needs.

**Conclusion:**

Short-video platforms have both supportive and stress-inducing effects on the psychological adjustment of parents of children with autism. These effects are shaped by interactions among platform algorithms, content presentation, family circumstances, and parental interpretation. Psychological adjustment involved recalibrating credibility, comparison, emotional responses, and caregiving decisions. Professionals should strengthen parents’ information-evaluation skills and digital health literacy, while platforms should improve content moderation, disclose creators’ qualifications and commercial interests, and regulate promotional content.

## Introduction

Autism spectrum disorder (ASD) is a neurodevelopmental condition characterized by differences in social communication and interaction and by restricted or repetitive patterns of behavior and interests. Raising a child with autism often involves sustained responsibilities related to intervention, education, daily care, and future planning. Parents of children with autism report substantially greater parenting stress than parents of typically developing children and parents of children with other developmental disabilities (Hayes and Watson, 2013). In China, parents also report unmet needs for autism-related knowledge, individualized caregiving skills, psychological and social support, and coordinated services (Li et al., 2025). As children develop, the challenges faced by families also shift, from decisions about intervention and schooling to social participation and longer-term care.

Psychological adjustment is especially relevant to autism caregiving because parents face demands that change across diagnosis, intervention, education, and long-term care. It involves ongoing cognitive, emotional, and behavioral responses to persistent stressors and changing circumstances (Sharpe and Curran, 2006; Stanton et al., 2007). In autism research, caregiver adjustment has been linked more specifically to parents’ psychological responses to their child’s diagnosis, although definitions and measures remain inconsistent (Clark-Whitney et al., 2026). Another study described parental adjustment in terms of acceptance, self-blame, and despair (Abd Latif et al., 2025). It differs from psychological distress, which mainly refers to negative states such as anxiety or depression, and from coping, which involves specific efforts to manage demands. Parental adaptation is broader, encompassing overall functioning and well-being under continuing caregiving demands (Higgins et al., 2023). Psychological adjustment thus captures changes in parents’ interpretations, emotions, expectations, and responses to caregiving over time.

Information and social support can be important resources within this process. Parents of children with autism seek guidance on diagnosis, intervention, education, everyday care, and long-term support, while also looking for reassurance and connection with families facing similar experiences (Li et al., 2025). When professional services or offline networks do not fully meet these needs, online environments provide additional routes to knowledge and support. A recent systematic review identified information seeking and engagement with parenting-related content as major features of parents’ social-media use (Mertens et al., 2024). Autism-specific online communities can also provide both informational and emotional support by allowing parents to exchange experiences and connect with others facing similar challenges (Roffeei et al., 2015). Chinese parents of children with autism likewise rely substantially on social-media sources, particularly WeChat public accounts and groups, when seeking autism-related information (Zhong and Han, 2026). Much of this literature, however, has focused on online communities or social media broadly, leaving the specific role of short-video platforms less clearly understood.

Short videos may be particularly relevant to autism caregiving because many intervention practices are procedural and context dependent. Strategies such as prompting, reinforcement, communication support, sensory regulation, and behavior management require parents to understand not only what to do but also how and when to respond. Audiovisual demonstrations can show actions, language, timing, and context simultaneously, while replay and saving functions allow parents to revisit content when needed. Evidence from professionally designed telehealth and video-modeling programs suggests that video-based instruction can support parents in learning autism-related intervention strategies (Bordini et al., 2020; Vismara et al., 2013). Everyday short-video use, however, extends beyond formal instruction to include explanations of autistic behaviors, rehabilitation and schooling information, parental experiences, and children’s developmental records. Karpinsky et al. (2025) found that prominent autism-related TikTok content focused mainly on lived experience and often offered limited actionable guidance. Short videos may therefore serve both as information sources and as experiential reference points through which parents interpret their child’s behavior, progress, and future possibilities.

Everyday short-video use differs markedly from structured video-based training. What parents encounter is shaped by their own viewing behavior as well as by platform recommendation algorithms, and the content comes from diverse sources, including clinicians, rehabilitation practitioners, parents, autistic individuals, institutions, influencers, and commercial accounts. Professional expertise, communicative purpose, and evidential quality therefore vary across videos. An analysis of 279 pediatric autism-related videos across Douyin, Bilibili, and Rednote (Xiaohongshu) found significant differences in video quality across platforms, with overall quality being moderate but information reliability remaining poor (Ou et al., 2026). Similar concerns have been reported internationally: highly viewed autism-related videos are not necessarily more accurate, and views or likes do not reliably distinguish accurate content from inaccurate or overgeneralized claims (Aragon-Guevara et al., 2025). Algorithmic recommendation further shapes what parents encounter. A search prompted by one concern can lead to repeated exposure to related material based on previous viewing and engagement. Such recommendation processes have been associated with technostress, perceived stress, and continued short-video use in health-related contexts (Wang et al., 2024). For parents already uncertain about their child’s development, repeated exposure may gradually shift attention from an immediate concern toward intervention claims, developmental outcomes, schooling difficulties, and longer-term care.

These features can generate both support and psychological pressure. Peer narratives and developmental records may reduce isolation and sustain hope, but they may also become standards against which parents judge their child’s progress or their own caregiving. Parenting-related social-media research suggests that relatable experiences can be supportive, whereas upward comparison may undermine perceived parenting competence and psychological well-being (Coyne et al., 2017; Egmose et al., 2022). For parents of children with autism, comparison may involve both children’s development and parental caregiving effort. Short-video narratives may intensify this process by compressing uneven developmental trajectories into brief accounts while omitting differences in baseline abilities, family resources, setbacks, and intervention conditions. Short-video platforms may therefore offer information and emotional connection while also contributing to confusion, self-doubt, comparison, and intervention-related anxiety.

Existing research has addressed online support for parents of children with autism, structured video-based parent training, and the quality of autism-related short-video content, but less is known about how these processes intersect in everyday platform use. It remains unclear how algorithmically curated short-video environments can simultaneously provide support and generate psychological pressure, or how parents adjust their interpretations, emotions, comparison standards, and caregiving decisions over time. Social support theory helps explain how guidance, reassurance, and shared experiences may provide informational, emotional, and appraisal support and buffer stress (Cohen and Wills, 1985; House, 1981), while social comparison theory explains how other children and caregivers may become reference points for evaluating development and caregiving (Festinger, 1954). The study therefore examined how Chinese parents of children with autism experience the supportive and stress-inducing effects of short-video platforms and how they psychologically adjust to these competing influences. The study had three objectives: (1) to explore how short-video platforms provide supportive experiences for Chinese parents of children with autism; (2) to examine how short-video content and platform features contribute to psychological pressure; and (3) to understand how parents adjust to the simultaneous supportive and stress-inducing influences of short-video use. These objectives were addressed through the following research questions:

RQ1: How do Chinese parents of children with autism experience the supportive effects of short-video platforms?

RQ2: How do Chinese parents of children with autism experience the stress-inducing effects of short-video platforms?

RQ3: How do Chinese parents of children with autism adjust to the simultaneous supportive and stress-inducing effects of short-video platforms?

## Materials and methods

### Study design

This study employed an exploratory qualitative design and conducted semi-structured, in-depth interviews to examine how short-video platforms shape the psychological adjustment of Chinese parents of children with autism. The interviews explored participants’ short-video use, experiences of informational and emotional support, social comparison, caregiving stress, and changes in parenting-related perceptions. Data were analyzed using thematic analysis, and the study was reported in accordance with the Consolidated Criteria for Reporting Qualitative Research guidelines (COREQ; Tong et al., 2007).

### Participants

Participants were recruited using purposive and snowball sampling, approaches commonly used in qualitative studies of parents of children with autism (e.g., Lamba et al., 2022). The researchers initially contacted four autism rehabilitation and family support organizations across four Chinese provinces, through which eligible parents were recruited. Recruitment advertisements were also posted on WeChat, Weibo, and Xiaohongshu. Participants who completed an interview were subsequently invited to refer other eligible parents. For the purposes of this study, short videos were defined as audiovisual content distributed through Douyin, Kuaishou, WeChat Channels, Xiaohongshu, Bilibili, Weibo Video, and similar platforms.

Participants were eligible if they: (1) had a child diagnosed with autism spectrum disorder by a qualified clinician and could provide a diagnostic report or relevant medical record; (2) held primary responsibility for the child’s daily care and rehabilitation-related decisions; (3) had used at least one of the specified short-video platforms and viewed autism-related content within the previous year; (4) could complete the interview in Mandarin Chinese; and (5) voluntarily agreed to participate and provided written informed consent. Primary caregiving responsibility was identified during eligibility screening based on participants’ self-identification as the family member mainly responsible for day-to-day care and substantially involved in rehabilitation-related decisions. Participants were excluded if they: (1) were not primarily responsible for the child’s care; (2) had no experience viewing autism-related short-video content; (3) were unable to complete the interview or declined audio recording; or (4) provided substantially incomplete interview data.

A total of 35 potential participants were contacted, of whom 11 were not included because they were ineligible, declined participation, or did not complete an interview. Data collection and preliminary analysis proceeded concurrently. In the later stages of recruitment, additional interviews mainly elaborated existing themes and no longer altered the developing thematic structure. Given the focused research questions, the specificity of the participant group, and the depth of the interviews, the final sample was considered sufficient to address the study aims (Hennink et al., 2017; Malterud et al., 2016). Ultimately, 24 parents, each from a different family, completed an interview. The sample comprised 13 mothers and 11 fathers, all of whom held primary caregiving responsibility. Participants were aged 29–48 years, and their children were aged 4–15 years. Of the 24 children, 22 were boys and 2 were girls. Time since the child’s diagnosis ranged from 1 to 11 years, and participants had viewed autism-related short-video content for 1–6 years. The platforms used most frequently were Douyin, WeChat Channels, Xiaohongshu, and Kuaishou. Participant characteristics are summarized in Table 1.

**TABLE 1:** Participant characteristics.

ID	Relationship	Participant age (years)	Education level	Child’s sex	Child’s age (years)	Time since diagnosis	Main platforms used	Duration of use
P01	Mother	34	Junior college	Male	8	4 years	Douyin, Xiaohongshu	3 years
P02	Father	40	Junior college	Male	11	6 years	Douyin, WeChat Channels	4 years
P03	Mother	35	Bachelor’s degree	Male	9	6 years	WeChat Channels, Xiaohongshu	5 years
P04	Mother	37	Junior college	Male	11	7 years	Douyin, WeChat Channels	4 years
P05	Father	42	Bachelor’s degree	Male	12	8 years	Douyin, Kuaishou	4 years
P06	Mother	29	Junior college	Female	4	2 years	Xiaohongshu, Douyin	1 year
P07	Father	38	Junior college	Male	10	6 years	Douyin, Kuaishou	5 years
P08	Mother	41	High school or below	Male	15	11 years	Douyin, WeChat Channels	5 years
P09	Mother	31	Junior college	Male	5	1 year	Xiaohongshu, Douyin	2 years
P10	Father	35	Bachelor’s degree	Male	7	2 years	WeChat Channels, Douyin	3 years
P11	Mother	40	Junior college	Male	13	9 years	Douyin, Kuaishou	6 years
P12	Father	33	Junior college	Male	6	2 years	Douyin, WeChat Channels	3 years
P13	Mother	29	Junior college	Female	5	2 years	Xiaohongshu, Douyin	2 years
P14	Father	44	Bachelor’s degree	Male	14	10 years	Douyin, WeChat Channels	4 years
P15	Mother	34	Junior college	Male	9	6 years	Douyin, Xiaohongshu	5 years
P16	Father	40	Master’s degree or above	Male	11	6 years	Xiaohongshu, Douyin	6 years
P17	Father	33	High school or below	Male	5	2 years	WeChat Channels, Douyin	2 years
P18	Father	32	Bachelor’s degree	Male	6	2 years	Douyin, Xiaohongshu	2 years
P19	Mother	39	Bachelor’s degree	Male	12	9 years	WeChat Channels, Xiaohongshu	2 years
P20	Father	37	Bachelor’s degree	Male	9	6 years	Douyin, Kuaishou	5 years
P21	Mother	33	Bachelor’s degree	Male	8	4 years	Douyin, Xiaohongshu	4 years
P22	Father	48	Junior college	Male	13	8 years	Douyin, WeChat Channels	5 years
P23	Mother	32	Bachelor’s degree	Male	7	1 year	Xiaohongshu, Douyin	1 year
P24	Mother	42	Junior college	Male	15	11 years	Douyin, Kuaishou	5 years

### Data collection

Individual semi-structured, in-depth interviews were conducted between February and May 2026 to explore participants’ experiences, meanings, and emotional responses in depth while maintaining a consistent set of topics across interviews (DeJonckheere and Vaughn, 2019; DiCicco-Bloom and Crabtree, 2006). Of the 24 interviews, 17 were conducted online via Tencent Meeting or WeChat voice calls and 7 were conducted face-to-face. Online interviews accommodated participants across different provinces and provided greater scheduling flexibility for parents with caregiving responsibilities, while face-to-face interviews were conducted according to participant preference. The same interview guide, probing approach, and recording procedures were used across both modes. Potential differences in rapport and contextual detail between online and face-to-face interviews were considered when interpreting the data.

The interview guide was developed from the study aims, social support theory, and relevant literature, with reference to established methodological guidance for developing semi-structured interview guides (Kallio et al., 2016). It was reviewed by two researchers with more than 5 years of qualitative research experience and three professionals working with families of children with autism. Two eligible parents participated in pilot interviews, after which several questions were reordered or reworded for clarity. Pilot data were excluded from the analysis. The complete interview guide, including main questions and probing prompts, is provided in Supplementary Appendix A.

Before the interview, participants completed a brief sociodemographic and platform-use form covering their age and sex, their child’s age, region of residence, time since diagnosis, duration of short-video use, and most frequently used platforms. Interviews explored short-video use, exposure to autism-related content, informational and emotional support, social comparison, caregiving stress, changes in parenting perceptions, and adverse platform-related experiences. Probing questions were used to clarify responses and elicit detailed accounts.

Interviews lasted 45–75 min (mean approximately 58 min) and were audio-recorded with consent. Field notes and reflexive memos were prepared after each interview. Recordings were transcribed verbatim within 48 h and checked against the audio for accuracy. Identifying information was removed, and participants were assigned codes P01–P24. Audio recordings, transcripts, and consent documents were stored on encrypted devices accessible only to the research team. Participants received an RMB 100 gift voucher after the interview.

### Data analysis

The data were analyzed using thematic analysis, following the approach developed by Braun and Clarke (2006). Anonymized interview transcripts were imported into NVivo 14.0 to organize the data, support coding, and document the analytic process. The analysis was primarily inductive, with social support theory and social comparison theory used as complementary frameworks for interpreting the findings (Cohen and Wills, 1985; Festinger, 1954; House, 1981). The analysis proceeded through six stages: (1) repeatedly reading the transcripts and listening to the recordings to develop familiarity with the data; (2) conducting open coding of material relevant to the research questions; (3) comparing common and divergent experiences across participants to generate candidate themes; (4) reviewing and refining these themes in relation to the original data; (5) defining and naming the themes and subthemes; and (6) selecting representative quotations to support the presentation of the findings. Two researchers met regularly to discuss their interpretations and address differences through further examination of the original transcripts. Changes in coding, analytic memos, and the rationale for revising the themes were documented throughout the analysis, creating an audit trail for the development of a coherent thematic structure grounded in the interview data.

### Ethical considerations

The study was approved by the Ethics Committee of Yunnan University and conducted in accordance with the Declaration of Helsinki (World Medical Association, 2025). All participants were adult parents; no children were directly involved in data collection. Written informed consent was obtained before the interviews, and participants were informed of confidentiality, voluntary participation, and their right to withdraw at any time.

## Results

Analysis of the interviews with 24 parents of children with autism generated three themes and 12 subthemes, which are summarized in Table 2.

**TABLE 2:** Themes and subthemes identified from the interviews.

Theme	Subtheme
Theme 1. Scrolling into Hope: Support Shaped by Content Form, Source, and Credibility	1.1 Audiovisual Demonstrations Lower Barriers to Understanding
	1.2 Algorithmic Recommendations Expand Information Access
	1.3 Parent Narratives Provide Experiential and Emotional Validation
	1.4 Longitudinal Records Provide a More Credible Basis for Hope
Theme 2. Scrolling into Anxiety: How Short-Video Features Contribute to Psychological Pressure	2.1 Decontextualized Intervention Advice Creates Uncertainty
	2.2 Algorithmic Recommendations Amplify Future-Oriented Anxiety
	2.3 Selective Success Narratives Distort Developmental and Caregiving Benchmarks
	2.4 Urgency Framing Intensifies Intervention Pressure
	2.5 Commercial Promotion Blurs Trust and Persuasion
Theme 3. Navigating Hope and Anxiety: Recalibrating Information, Emotion, and Comparison	3.1 Recalibrating Credibility: Moving Beyond Popularity-Based Judgments
	3.2 Establishing Emotional and Comparison Boundaries
	3.3 Integrating Online Experiences with Professional and Child-Specific Knowledge

Theme 1. Scrolling into Hope: Support Shaped by Content Form, Source, and Credibility

Participants valued short-video content differently according to its form, source, and perceived credibility. Demonstrations supported practical understanding, parent narratives offered experiential validation, and longitudinal records provided a more credible basis for hope, while algorithmic recommendations broadened access to relevant information.

*Subtheme 1.1 Audiovisual Demonstrations Lower Barriers to Understanding*

Participants described autism-related assessment, behavioral intervention, and home-based training as difficult to understand through brief clinical explanations or written materials alone. Videos made these practices more concrete by showing how instructions, waiting periods, prompts, and reinforcement were delivered. Pausing and replaying allowed parents to review specific procedures at their own pace.

*“Teachers used to tell me to prompt, wait, and reinforce. I understood the terms, but I did not know exactly how to do them. The videos demonstrate when to speak, when to stop, and how long to wait for the child. I can watch them repeatedly and then try the techniques myself.”* (P06)

The usefulness of these videos lay partly in making procedural knowledge observable. Audiovisual content also helped parents reinterpret their children’s sensory, communication, and emotional responses. One mother reported that a simulation of sensory overload helped her recognize her child’s ear-covering as a response to genuine discomfort rather than deliberate disobedience (P15). Some parents also shared such videos with spouses or grandparents to build a more consistent understanding of the child’s needs.

***Subtheme 1.2 Algorithmic Recommendations Expand Information Access***

Most participants initially encountered autism-related content through purposeful searches. As they watched, liked, or saved videos, recommendations introduced topics and services they had not previously known to seek. This was particularly useful shortly after diagnosis, when parents were often unsure what information they needed.

Recommendations gradually broadened information seeking from immediate language and behavioral concerns to home-based training, school preparation, public support, and long-term care. *“It was as though the platform was telling me step by step that, apart from the speech problem, there were many other issues that we needed to prepare for in advance.”* (P18)

Recommended content also introduced parents to online lectures, rehabilitation subsidies, school admissions information, and parent-training programs. For participants with limited access to professional resources, this discovery function opened routes to information that were otherwise difficult to obtain (P12). Participants valued recommendation primarily as a way of discovering information.

***Subtheme 1.3 Parent Narratives Provide Experiential and Emotional Validation***

The source of content shaped the type of support parents experienced. Parent-created narratives were valued for the recognizability of lived experience. Accounts of diagnosis, intervention setbacks, social misunderstanding, and caregiving exhaustion allowed participants to recognize their own emotions in the experiences of others.

*“One mother in a video also talked about feeling exhausted and sometimes losing her temper with her child. I actually felt relieved after watching it. I realized that I was not the only one who sometimes broke down and that having one breakdown did not mean I was a bad mother.”* (P21)

Comment sections provided a similar sense of shared experience, even among parents who rarely posted themselves (P17). Yet experiential similarity did not always bring reassurance. Content reflecting situations that parents personally feared could also evoke distress (P09). Relatability therefore increased the emotional relevance of peer content, although its emotional effect was not always positive.

***Subtheme 1.4 Longitudinal Records Provide a More Credible Basis for Hope***

Profile archives and serialized posts allowed parents to follow the same child or family over time. Participants placed greater trust in records that showed gradual progress, setbacks, and continuing difficulties than in promotional content centered on dramatic outcomes. Credibility was associated with developmental context and consistency over time rather than positive outcomes alone.

*“I have followed one family for many years. The child did not suddenly become better; he gradually learned to express himself and dress independently. Watching that long-term record makes me feel that even though our progress is slow, it does not mean there is no possibility for improvement in the future.”* (P08)

Seeing other families experience periods of limited progress before later improvement was reassuring when parents felt their own children had stalled (P20). Longitudinal records also encouraged more individualized definitions of progress, including expressing discomfort, waiting briefly, reducing emotional outbursts, and becoming more independent in daily life (P01).

Across the subthemes, supportive experiences were associated with content that was practically useful, experientially relatable, contextually informative, and perceived as credible.

**Theme 2. Scrolling into Anxiety: How Short-Video Features Contribute to Psychological Pressure**

Participants’ anxiety was shaped by how autism-related content was presented and recommended. Decontextualized advice, repeated recommendations, selective success narratives, urgency-based messaging, and commercial promotion contributed to uncertainty, comparison, and intervention-related pressure.

***Subtheme 2.1 Decontextualized Intervention Advice Creates Uncertainty***

The brevity of short videos often reduced complex intervention issues to simplified methods and visible outcomes. Information needed to judge whether a strategy was appropriate (such as the child’s age, baseline abilities, individual differences, and conditions for implementation) was frequently absent. One father noted that videos sometimes recommended behavioral strategies without explaining which children or age groups they were intended for (P05). Participants therefore considered both the technique presented and whether enough contextual information was provided to judge its relevance to their own child.

Conflicting recommendations further complicated these judgments: *“Some people say that parents must seize the critical period, while others say that too much training can harm the child. The more I watch, the less I know what to do. It feels as though either choice could be wrong.”* (P13)

When information lacked sufficient context, individual experiences could be interpreted as broadly applicable advice. This left some parents uncertain about which recommendations to trust and concerned that an inappropriate choice might adversely affect their child’s development.

***Subtheme 2.2 Algorithmic Recommendations Amplify Future-Oriented Anxiety***

Personalized recommendations helped parents discover relevant information, but repeated exposure could also narrow attention around particular concerns. Searches about immediate language or behavioral difficulties sometimes led to videos about developmental regression, barriers to inclusive education, long-term care, and future arrangements when parents could no longer provide care.

*“I originally wanted to find out how to train my child to respond to his name. Later, the platform kept recommending videos about older children being unable to attend school and what would happen to them when their parents became old. Those issues are still far away from us, but watching them repeatedly made me afraid in advance.”* (P23)

The effect was often cumulative rather than tied to a single video. Autoplay and infinite scrolling made disengagement difficult, and some parents continued browsing despite anxiety and sleep disruption because they feared missing useful information (P02). Participants described a reinforcing pattern in which concern prompted further searching, longer engagement produced more similar recommendations, and repeated exposure made distant risks feel increasingly salient and immediate (P10). Thus, concerns that were not yet part of parents’ current caregiving situation could become emotionally salient through repeated recommendation.

***Subtheme 2.3 Selective Success Narratives Distort Developmental and Caregiving Benchmarks***

Short videos often highlighted visible progress while leaving stagnation, regression, and family investment less visible. Editing, music, and coherent storytelling could compress months or years of uneven development into apparently straightforward success narratives. In contrast to longitudinal accounts that included setbacks and continuing difficulties, these selectively presented stories offered less context for judging how representative the portrayed progress actually was.

When parents saw similarly aged children communicating verbally, attending mainstream schools, or completing daily activities independently, they compared these achievements with their own children:

*“When I saw a child who was about the same age as mine already having conversations, my first reaction was not happiness for that child. I wondered why my own child still could not do it. After watching the video, I felt that perhaps we had fallen too far behind.”* (P04)

Comparison also extended to parental performance. Videos portraying highly structured daily training and consistently patient caregiving led some participants to question whether they were investing sufficient time and effort, and slower progress was sometimes attributed to perceived shortcomings in their own caregiving (P21). Likes, comments, and follower counts could further increase the perceived credibility and representativeness of selected success stories, even when important developmental and family context was missing (P11). Comparison therefore operated at two levels: parents evaluated both their child against other children and themselves against other caregivers.

***Subtheme 2.4 Urgency Framing Intensifies Intervention Pressure***

Participants frequently encountered content emphasizing narrow intervention windows and severe consequences, using phrases such as “Do not miss the critical period” or “It will be too late if parents do not act.” Such messages created pressure for rapid and continuous intervention, even when children were already receiving regular support. One mother described feeling repeatedly reminded that she could not reduce or pause intervention efforts (P03).

Music, captions, and dramatic before-and-after contrasts further intensified fear, guilt, and self-doubt: *“Some videos begin by saying, ‘One mistake by a parent could delay a child for life,’ accompanied by very tense music. Although I know it may be exaggerated, I still wonder whether I have done something wrong.”* (P19)

Repeated exposure to urgency-based content made gradual or uneven development more difficult for some parents to tolerate. When progress was not immediately visible, they worried that they had missed an optimal intervention window or needed to change methods quickly (P16). Claims framed primarily through fear and urgency were therefore difficult for parents to evaluate when their applicability to individual children was unclear.

***Subtheme 2.5 Commercial Promotion Blurs Trust and Persuasion***

The source and purpose of short-video content became especially important when educational information, shared experience, and commercial promotion overlapped. Some parent creators first established trust by discussing experiences familiar to other families and later promoted intervention programs, training materials, nutritional products, or institutional services. In these cases, trust grounded in shared caregiving experience could extend to commercial recommendations.

One father purchased an online course promoted by a parent creator whom he believed had “walked the same path.” When the course did not produce the advertised results, he initially questioned his own implementation rather than the recommendation itself: *“I wondered whether my child had failed to achieve the results shown in the video because I had not persisted enough.”* (P07)

Institutional accounts presented a different credibility cue. Their professional appearance could lend authority to messages framing children’s difficulties as problems requiring immediate paid intervention. For some participants, this increased pressure to purchase courses, assessments, or training materials out of concern that refusing a recommended service might delay their child’s development (P06). Parents therefore had to consider both whether information was credible and whether commercial interests shaped how it was presented.

Across the subthemes, psychological pressure was linked to content that lacked context, repeated exposure to risk-related information, selective success narratives, urgency-based framing, and blurred commercial intent.

**Theme 3. Navigating Hope and Anxiety: Recalibrating Information, Emotion, and Comparison**

Participants described changes over time in how they engaged with autism-related short videos. Psychological adjustment involved becoming more selective in judging credibility, managing emotional responses, interpreting comparisons, and incorporating online information into caregiving decisions, while recognizing that short videos could provide support and also amplify uncertainty and pressure.

***Subtheme 3.1 Recalibrating Credibility: Moving Beyond Popularity-Based Judgments***

Some participants initially interpreted high view counts, large numbers of likes, and repeated recommendations as indicators that content was important or trustworthy. With continued use of short-video platforms, however, they became aware that recommendation frequency was largely shaped by viewing history, engagement behavior, and platform algorithms rather than by professional evidence or informational quality.

*“I used to think that if the platform kept recommending something, it must be especially important. Later, I realized that it was recommended repeatedly simply because I had spent more time watching that type of video. It did not mean that the information was necessarily correct.”* (P10)

This shift changed how parents evaluated autism-related content. Participants became more cautious about absolute claims, exaggerated before-and-after comparisons, emotionally charged headlines, and unclear commercial intentions. Some learned to interpret statements such as “It will be too late if you do not do this” as attention-oriented messages rather than universally applicable guidance (P24). Adjustment therefore involved moving from relying on visible platform signals toward more critical evaluation of content relevance, evidence, and applicability to their own child.

***Subtheme 3.2 Establishing Emotional and Comparison Boundaries***

Participants also described changes in how they managed emotional reactions triggered by short-video content. Some limited viewing time, unfollowed accounts that repeatedly increased anxiety, stopped browsing when distressed, or avoided autism-related content before bedtime.

*“I used to keep scrolling before going to sleep, and the more I watched, the more alert I became. Now, I have a rule that I do not watch autism-related videos after 10 p.m.; otherwise, I feel depressed throughout the following day.”* (P22)

Adjustment also involved changes in comparison standards. Rather than continuously comparing their children with selectively presented online cases, some parents gradually focused more on their own child’s previous development and individual progress. As one mother explained: *“I used to compare my child with children in the videos and wonder why they could do things that he still couldn’t. Now I try to look at how he is doing compared with a few months ago. Even a small improvement feels meaningful to me.”* (P19)

This shift reflected a move from externally defined developmental benchmarks toward more individualized interpretations of progress.

***Subtheme 3.3 Integrating Online Experiences with Professional and Child-Specific Knowledge***

Participants increasingly distinguished between experiential information from other families and professional guidance. While parent experiences provided practical ideas and emotional reassurance, they were not considered directly transferable because children differed in age, abilities, developmental trajectories, and family circumstances.

Some parents began evaluating online suggestions in relation to their own child’s needs and consulting clinicians or rehabilitation professionals before making important caregiving decisions (P07). Online content was therefore incorporated as a supplementary source of knowledge rather than a replacement for professional assessment.

*“I still feel worried when I see a video that makes me anxious, but I no longer immediately enroll my child in a course or assume that I have failed. I pause first and consider whether this is genuinely a problem that we need to address.”* (P14)

Psychological adjustment did not mean that anxiety disappeared. Instead, parents became better able to distinguish concerns arising from their child’s actual needs from worries intensified by platform content, creating greater distance between emotional reactions and immediate decisions.

Across these accounts, parents’ adjustment reflected a process of recalibration: they continued to use short videos for information and support while developing more selective standards for judging credibility, managing emotions, and making caregiving decisions.

## Discussion

Short-video platforms occupied an ambivalent place in parents’ caregiving experiences. Previous research has documented informational and emotional support in online autism communities (Roffeei et al., 2015), information seeking through parenting-related social media (Mertens et al., 2024), social comparison among parents online (Coyne et al., 2017; Egmose et al., 2022), and uneven quality in autism-related digital content (Aragon-Guevara et al., 2025). The present findings bring these processes together within the same short-video environment. Content that clarified intervention practices, reduced uncertainty, or reflected familiar caregiving experiences could provide reassurance and strengthen confidence. Continued engagement could also expose parents to conflicting advice, selective developmental narratives, future-oriented risks, and commercial persuasion. Support and psychological pressure therefore emerged through interconnected platform experiences, shaped by content form, source, credibility, recommendation processes, and parents’ caregiving circumstances. This helps explain why the psychological effects of short-video use cannot be captured adequately by classifying content as simply supportive or stressful.

A key contribution concerns the way short videos reshape parents’ access to autism-related knowledge. Audiovisual demonstrations transformed practices such as prompting, reinforcement, and home-based training into observable sequences of action, making specialized knowledge easier to understand and revisit. This adds an important dimension to research on informational support: the usefulness of information depends partly on how knowledge is communicated. Yet easier access created a parallel challenge of evaluation. Brief videos often omitted children’s baseline abilities, intervention goals, individual differences, and conditions of applicability, while engagement indicators provided little assurance of informational accuracy (Aragon-Guevara et al., 2025). Parents consequently faced an access–evaluation tension. Short videos lowered the barrier to understanding specialized knowledge while increasing the need to judge its credibility and relevance with limited professional guidance (Zhao et al., 2025). Content source also shaped perceived credibility and usefulness, with professional accounts valued for expertise and parent-created content for shared caregiving experience (Brown et al., 2024). Parent narratives gained value through experiential similarity, whereas longitudinal records that included setbacks and uneven progress were often perceived as more credible than highly polished success stories. Informational and emotional support in short-video environments therefore depended strongly on context, source, and perceived credibility.

A second contribution lies in the interaction between algorithmic recommendation and caregiving responsibility. Research on health-related short-video use has shown that recommendation-algorithm technostress can shape perceived stress and continued platform engagement (Wang et al., 2024), while doomscrolling has been associated with greater psychological distress and poorer well-being (Satici et al., 2023). The present findings identify a caregiving-specific process behind continued exposure. Parents often began with an immediate concern about language, behavior, or intervention, but subsequent recommendations shifted attention toward schooling difficulties, long-term dependence, financial burden, and future care (Han et al., 2025). Repeated exposure made these distant possibilities emotionally salient before they became part of the family’s current situation. Disengagement could itself feel risky because parents feared overlooking information or intervention opportunities that might benefit their child. Uncertainty therefore encouraged further searching; continued engagement generated increasingly similar recommendations; and repeated exposure strengthened anticipatory concern. Algorithmic personalization became psychologically consequential through its interaction with parents’ perceived responsibility to remain informed about every possible opportunity or risk.

The findings also add greater specificity to social comparison processes in digital parenting environments. Previous studies have shown that social-media comparison can affect parental competence and psychological well-being (Coyne et al., 2017; Egmose et al., 2022; Glatz et al., 2026). In the present study, comparison occurred at two interconnected levels. Parents evaluated their child’s communication, schooling, and independence against other children while also evaluating their own effort, patience, and intervention investment against other caregivers. These reference points were shaped by platform presentation. Editing could compress prolonged and uneven developmental trajectories into coherent stories of success, while views, likes, and follower counts could increase the apparent credibility and representativeness of selected cases. Comparison standards were therefore partly produced through platform visibility and selective presentation (Lee et al., 2025). This is especially important in autism caregiving, where developmental trajectories and intervention contexts vary considerably. When highly visible cases become implicit benchmarks, differences in baseline ability, family resources, and intervention conditions can recede from view, increasing self-doubt and self-blame. The findings therefore show that social comparison targets in short-video environments are actively shaped by the architecture and visibility logic of the platform.

The clearest contribution concerns psychological adjustment. Participants described changes in the standards through which they interpreted and responded to short-video content. Popularity and repeated recommendation gradually became less persuasive as indicators of credibility; comparison shifted toward the child’s own developmental trajectory; emotional reactions were given more distance before being translated into intervention or purchasing decisions; and online experiences were increasingly assessed alongside professional guidance and the child’s individual circumstances. This pattern gives psychological adjustment a more specific meaning in algorithmically curated environments. Adjustment involved recalibrating credibility, comparison standards, emotional exposure, and caregiving decisions as parents navigated competing sources of reassurance and pressure. From a social comparison perspective, parents moved toward more individualized developmental benchmarks and reduced reliance on platform-supplied reference points. From a social support perspective, they became more selective in combining experiential knowledge, professional guidance, and child-specific information. This is consistent with evidence that Chinese parents of children with autism use multiple interpersonal and online sources according to particular caregiving needs (Zhong and Han, 2026). Psychological adjustment therefore involved a changing relationship with digital information: anxiety remained present, but parents increasingly altered how that anxiety was interpreted and how strongly it influenced immediate action.

## Theoretical and practical implications

Theoretically, the findings connect social support, social comparison, and online health-information research through the concept of platform-mediated psychological adjustment. Short-video platforms shape more than access to support or opportunities for comparison. Audiovisual formats influence what parents can understand; recommendation systems influence what concerns become salient; visibility metrics influence what appears credible or representative; and heterogeneous content sources influence how professional authority, lived experience, and commercial intent are interpreted (Brown et al., 2024). Social support theory therefore helps explain how informational, emotional, and appraisal resources are obtained and evaluated (Cohen and Wills, 1985; House, 1981; Han et al., 2026), while social comparison theory helps explain how platform-mediated reference points influence judgments of child development and parental performance (Festinger, 1954). The present findings link these processes through psychological adjustment, understood as continuing recalibration of credibility, comparison, emotional exposure, and caregiving judgment.

Practically, clinicians, rehabilitation practitioners, and social workers should treat parents’ short-video use as part of the broader caregiving information environment. Guidance should address how to assess creator qualifications, identify missing contextual information, recognize commercial interests, and distinguish platform popularity from evidential credibility. Professionals can also help parents interpret developmental progress against their child’s individual trajectory and discuss anxiety triggered by future-oriented content before it shapes intervention decisions. Platforms have a parallel responsibility to improve disclosure of professional qualifications and commercial relationships, restrict unsupported or fear-based treatment claims, and strengthen access to contextualized, evidence-informed autism information (Ononuju and Ujari, 2025). Parent organizations and healthcare institutions may be especially valuable when they provide realistic developmental narratives that include periods of limited progress alongside improvement.

## Limitations and future research

This study has several limitations. The sample was small and consisted only of parents with experience using short-video platforms who were recruited mainly through autism rehabilitation organizations and online channels, which may limit the transferability of the findings. The study also relied on retrospective self-reports and did not collect objective data on viewing histories, screen time, or algorithmically recommended content. In addition, differences across platforms were not systematically examined, and the use of a single interview with each participant prevented the study from capturing changes over time. The analysis focused only on parents’ perspectives and did not include children with autism, clinicians, rehabilitation practitioners, or content creators. Future research should therefore use more diverse samples, longitudinal and cross-platform designs, objective platform-use data, and multi-stakeholder or quantitative approaches to examine the mechanisms linking short-video use with parental psychological adjustment.

## Conclusion

This study shows that short-video platforms have become an important part of the information seeking, emotional experiences, and caregiving decisions of Chinese parents of children with autism. Algorithms, visual narratives, social interaction, and commercial content can create both support and psychological pressure by shaping what parents encounter, compare, and perceive as credible. Psychological adjustment involved gradually recalibrating credibility judgments, comparison standards, emotional responses, and caregiving decisions as parents gained experience with short-video platforms. Addressing misinformation, algorithmic pressure, and commercial persuasion requires shared responsibility among professionals, healthcare and parent organizations, and platforms to improve content quality, transparency, and access to reliable support.

## Data Availability

The raw data supporting the conclusions of this article will be made available by the authors, without undue reservation.
