# Supplementary material for: Scrolling into hope and anxiety: a qualitative study of how short-video platforms shape psychological adjustment among Chinese parents of children with autism
Source: Front Psychol. 2026 Sep 9;17:1952061. doi: 10.3389/fpsyg.2026.1952061 (PMC13597810; doi:10.3389/fpsyg.2026.1952061)
Supplement: Supplementary file 1 [file Supplementary_file_1.docx]

**Appendix A**

**Semi-Structured Interview Guide**

**Opening Statement**

Hello, and thank you very much for taking part in this interview. We would like to learn about your experiences of using short-video platforms and encountering autism-related content. There are no right or wrong answers. Please describe your experiences based on your own circumstances.

The information collected will be used solely for research purposes. Any information that could identify you or your child will be anonymized. With your consent, this interview will be audio-recorded. You may decline to answer any question, pause the interview, or withdraw at any time. Have you understood the information above, and do you agree to begin the interview and allow it to be recorded?

***I. Caregiving Background and Platform Use***

1. Could you tell me about your child’s current situation and the caregiving responsibilities you usually undertake?

2. How did you first encounter short videos related to autism?

3. Could you describe your current use of short-video platforms?

4. What types of autism-related content do you usually encounter or follow?

***II. Viewing Experiences and Their Influence***

5. Could you recall an autism-related short video that left a particularly strong impression on you?

6. What thoughts or feelings did it evoke?

7. Has this type of content changed how you understand your child’s behavior, abilities, or needs?

8. Have short videos influenced your home-based training, intervention choices, educational arrangements, or other caregiving decisions?

9. When viewing videos about other children with autism or their families, how do you respond to their experiences? (Possible probe: Do you ever compare your child’s development or your own caregiving with what you see? How does this affect you?)

10. What do the stories, comments, or interactions of other parents mean to you?

***III. Information Evaluation and Platform Experiences***

11. Have you encountered autism-related information that was difficult to assess, contradictory, or made you uncertain?

12. How do you decide whether a short video is credible and appropriate for your child? (Possible probes: Do the creator’s identity, professional qualifications, views, likes, comments, or other features affect your judgment?)

13. Have you noticed any patterns or changes in the autism-related content recommended to you after you watch, search for, like, or save certain videos? (Possible probe: How do repeated recommendations affect what you continue watching or how you feel?)

14. How do you view information about courses, service providers, training tools, or products presented in short videos?

***IV. Changes in Use and Psychological Adjustment***

15. As you have continued using these platforms, has the way you view and respond to autism-related short videos changed? (Possible probes: Have you changed how you evaluate information, manage emotional reactions, compare your child with others, or decide whether to act on advice from videos?)

16. Overall, what role do short videos play in your caregiving life?

17. Is there anything important about your experiences of using short videos and caring for your child that we have not asked about?

**Closing Statement**

Thank you very much for sharing your experiences. Your account will help us better understand how short videos influence the information seeking, emotional experiences, and caregiving decisions of parents of children with autism. After the interview, the recording will be transcribed and anonymized, and all data will be used solely for this study. Do you have any questions for the research team?
